# Dibromidobis(pyridine-3-carbonitrile-κ*N* ^1^)zinc(II)

**DOI:** 10.1107/S1600536810051342

**Published:** 2010-12-18

**Authors:** Reza Ghiasi

**Affiliations:** a Department of Chemistry, Basic Science Faculty, East Tehran, Islamic Azad University, Qiam Dasht Branch, Tehran, Iran

## Abstract

In the title compound, [ZnBr_2_(C_6_H_4_N_2_)_2_], the Zn^II^ atom is four coordinated in a slightly distorted tetra­hedral fashion by two pyridine N atoms and two Br^−^ anions. π–π inter­actions between adjacent pyridine rings [centroid–centroid distance = 3.6229 (19) Å] are the main factor controlling the packing and are effective in the stabilization of the crystal structure.

## Related literature

For related structures, see: Li *et al.* (2004 ▶); Steffen & Palenik (1976 ▶, 1977 ▶).
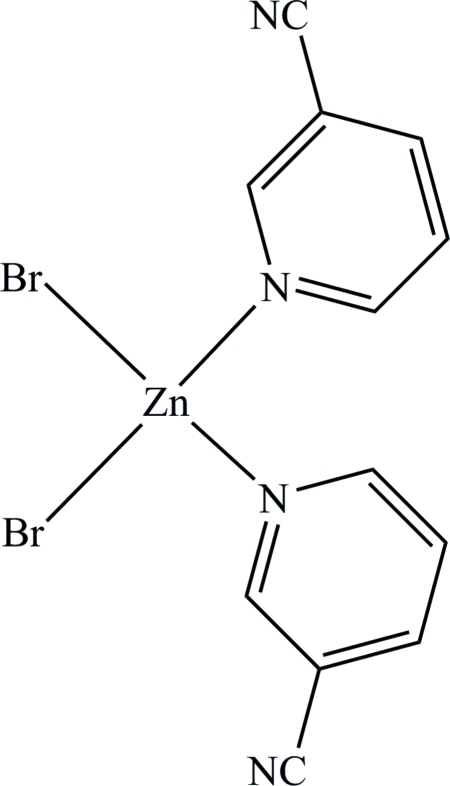


## Experimental

### 

#### Crystal data

[ZnBr_2_(C_6_H_4_N_2_)_2_]
*M* *_r_* = 433.41
Orthorhombic, 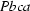

*a* = 8.5600 (4) Å
*b* = 14.5379 (5) Å
*c* = 23.3751 (9) Å
*V* = 2908.9 (2) Å^3^
*Z* = 8
Mo *K*α radiation
μ = 7.17 mm^−1^
*T* = 120 K
0.40 × 0.30 × 0.22 mm

#### Data collection

Bruker SMART CCD area-detector diffractometer
Absorption correction: multi-scan (*SADABS*; Bruker, 2001 ▶) *T*_min_ = 0.071, *T*_max_ = 0.210
12287 measured reflections
3876 independent reflections
2842 reflections with *I* > 2σ(*I*)
*R*_int_ = 0.053

#### Refinement

*R*[*F* ^2^ > 2σ(*F* ^2^)] = 0.035
*wR*(*F* ^2^) = 0.070
*S* = 0.97
3876 reflections
172 parameters
H-atom parameters constrained
Δρ_max_ = 0.82 e Å^−3^
Δρ_min_ = −0.50 e Å^−3^

### 

Data collection: *SMART* (Bruker, 2001 ▶); cell refinement: *SAINT* (Bruker, 2001 ▶); data reduction: *SAINT*; program(s) used to solve structure: *SHELXS97* (Sheldrick, 2008 ▶); program(s) used to refine structure: *SHELXL97* (Sheldrick, 2008 ▶); molecular graphics: *ORTEP-3 for Windows* (Farrugia, 1997 ▶); software used to prepare material for publication: *WinGX* (Farrugia, 1999 ▶).

## Supplementary Material

Crystal structure: contains datablocks global, I. DOI: 10.1107/S1600536810051342/bt5431sup1.cif

Structure factors: contains datablocks I. DOI: 10.1107/S1600536810051342/bt5431Isup2.hkl

Additional supplementary materials:  crystallographic information; 3D view; checkCIF report

## Figures and Tables

**Table:** 

N1—Zn1	2.061 (3)
N3—Zn1	2.072 (3)
Zn1—Br2	2.3369 (5)
Zn1—Br1	2.3471 (5)

**Table:** 

N1—Zn1—N3	100.85 (11)
N1—Zn1—Br2	111.60 (8)
N3—Zn1—Br2	108.98 (8)
N1—Zn1—Br1	105.62 (8)
N3—Zn1—Br1	105.82 (8)
Br2—Zn1—Br1	121.841 (19)

**Fig. 1.**
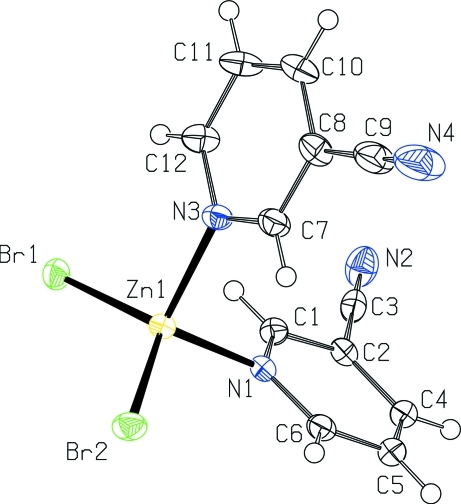
The molecular structure of the title molecule, with the atom-numbering scheme. Displacement ellipsoids are drawn at the 50% probability level.

**Fig. 2.**
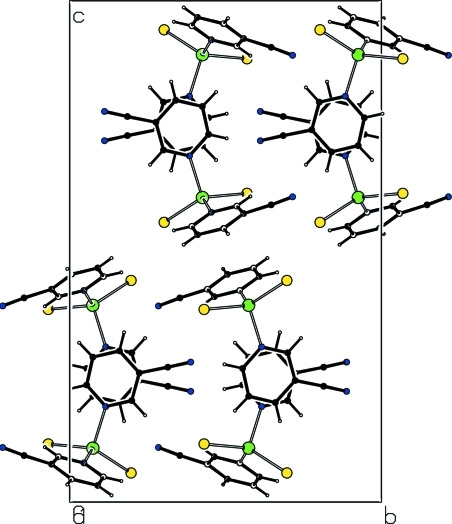
Packing diagram of the title compound with view along the a-axis.

## supplementary crystallographic information

### Comment

Several complexes  with the formula [*MX*_2_*L*_2_], such as [ZnCl_2_(py)_2_], (Steffen & Palenik, 1976), [ZnCl_2_(4-cypy)_2_], (Steffen & Palenik, 1977), [CuBr_2_(3-Cypy)_2_], (Li *et al.* 2004), [where py is pyridine, 4-cypy is 4-cyanopyridine and 3-cypy is 3-cyanopyridine] have been synthesized and characterized by single-crystal X-ray diffraction methods. The molecular structure of the title compound is shown in Fig. 1. The Zn^II^ atom is four-coordinated in a slightly distorted tetrahedral configuration by two N atoms from two pyridine rings and two Br^-^ anions. The Zn—Br and Zn—N bond distances and angles (Table 1) are within normal ranges. It seems that π-π interactions between adjacent pyridine rings [centroid···centroid distance of 3.6229 (19) Å, symmetry codes: 1 - *x*,-*y*,1 - *z*] are the main factor controlling the packing and are effective in the stabilization of the crystal structure.

### Experimental

Zinc(II) bromide (0.45 gr, 2 mmol) was disolved in methanol (10 ml) and the solution was mixed with a methanolic solution (10 ml) of 3-pyridinecarbonitrile (0.42 g, 4 mmol). This solution was left to evaporate slowly at room temperature. After one week, colorless block shaped crystals of the title compound were isolated (yield 0.66 g, 75.9%, m.p. < 580 K).

### Refinement

All H atoms were positioned geometrically, with C—H = 0.96Å atoms and constrained to ride on their parent atoms, with *U*_iso_(H)=1.2*U*_eq_(C).

### Crystal data

**Table:**

[ZnBr_2_(C_6_H_4_N_2_)_2_]	*D*_x_ = 1.979 Mg m^−^^3^
*M**_r_* = 433.41	Mo *K*α radiation, λ = 0.71073 Å
Orthorhombic, *P**b**c**a*	Cell parameters from 12287 reflections
*a* = 8.5600 (4) Å	θ = 2.9–29.1°
*b* = 14.5379 (5) Å	µ = 7.17 mm^−^^1^
*c* = 23.3751 (9) Å	*T* = 120 K
*V* = 2908.9 (2) Å^3^	Prism, colorless
*Z* = 8	0.4 × 0.3 × 0.22 mm
*F*(000) = 1664	

### Data collection

**Table:**

Bruker SMART CCD area-detector diffractometer	2842 reflections with *I* > 2σ(*I*)
graphite	*R*_int_ = 0.053
φ and ω scans	θ_max_ = 29.1°, θ_min_ = 2.9°
Absorption correction: multi-scan (*SADABS*; Bruker, 2001)	*h* = −11→9
*T*_min_ = 0.071, *T*_max_ = 0.210	*k* = −19→16
12287 measured reflections	*l* = −26→31
3876 independent reflections	

### Refinement

**Table:**

Refinement on *F*^2^	Primary atom site location: structure-invariant direct methods
Least-squares matrix: full	Secondary atom site location: difference Fourier map
*R*[*F*^2^ > 2σ(*F*^2^)] = 0.035	Hydrogen site location: inferred from neighbouring sites
*wR*(*F*^2^) = 0.07	H-atom parameters constrained
*S* = 0.97	*w* = 1/[σ^2^(*F*_o_^2^) + (0.0331*P*)^2^] where *P* = (*F*_o_^2^ + 2*F*_c_^2^)/3
3876 reflections	(Δ/σ)_max_ = 0.009
172 parameters	Δρ_max_ = 0.82 e Å^−^^3^
0 restraints	Δρ_min_ = −0.50 e Å^−^^3^

### Special details

**Table:**

Geometry. All e.s.d.'s (except the e.s.d. in the dihedral angle between two l.s. planes) are estimated using the full covariance matrix. The cell e.s.d.'s are taken into account individually in the estimation of e.s.d.'s in distances, angles and torsion angles; correlations between e.s.d.'s in cell parameters are only used when they are defined by crystal symmetry. An approximate (isotropic) treatment of cell e.s.d.'s is used for estimating e.s.d.'s involving l.s. planes.
Refinement. Refinement of *F*^2^ against ALL reflections. The weighted *R*-factor *wR* and goodness of fit *S* are based on *F*^2^, conventional *R*-factors *R* are based on *F*, with *F* set to zero for negative *F*^2^. The threshold expression of *F*^2^ > σ(*F*^2^) is used only for calculating *R*-factors(gt) *etc*. and is not relevant to the choice of reflections for refinement. *R*-factors based on *F*^2^ are statistically about twice as large as those based on *F*, and *R*- factors based on ALL data will be even larger.

### Fractional atomic coordinates and isotropic or equivalent isotropic displacement parameters (Å^2^)

**Table:**

	*x*	*y*	*z*	*U*_iso_*/*U*_eq_	
C1	0.5292 (4)	−0.0361 (2)	0.40962 (14)	0.0200 (7)	
H1	0.469	−0.0795	0.3904	0.024*	
C2	0.6824 (4)	−0.0582 (2)	0.42453 (14)	0.0200 (7)	
C3	0.7438 (4)	−0.1464 (2)	0.40720 (15)	0.0233 (7)	
C4	0.7723 (4)	0.0060 (2)	0.45419 (15)	0.0222 (7)	
H4	0.8741	−0.0077	0.4651	0.027*	
C5	0.7070 (4)	0.0900 (2)	0.46690 (15)	0.0231 (7)	
H5	0.7643	0.1342	0.4866	0.028*	
C6	0.5542 (4)	0.1082 (2)	0.44994 (14)	0.0202 (7)	
H6	0.5112	0.1655	0.4581	0.024*	
C7	0.3503 (4)	0.1991 (2)	0.29772 (15)	0.0252 (7)	
H7	0.3606	0.2419	0.327	0.03*	
C8	0.3943 (4)	0.2234 (2)	0.24242 (16)	0.0276 (8)	
C9	0.4489 (5)	0.3149 (3)	0.23096 (18)	0.0382 (10)	
C10	0.3819 (4)	0.1597 (3)	0.19849 (15)	0.0286 (7)	
H10	0.4128	0.1745	0.1615	0.034*	
C11	0.3233 (5)	0.0745 (3)	0.21094 (16)	0.0307 (8)	
H11	0.3131	0.0304	0.1823	0.037*	
C12	0.2792 (4)	0.0545 (2)	0.26661 (15)	0.0264 (7)	
H12	0.238	−0.0033	0.2746	0.032*	
N1	0.4666 (3)	0.04558 (19)	0.42210 (12)	0.0188 (6)	
N2	0.7925 (3)	−0.2151 (2)	0.39115 (15)	0.0320 (7)	
N3	0.2937 (3)	0.11564 (19)	0.30954 (12)	0.0203 (6)	
N4	0.4901 (6)	0.3883 (3)	0.22082 (17)	0.0585 (12)	
Zn1	0.24508 (4)	0.07419 (2)	0.392462 (16)	0.01808 (9)	
Br1	0.11976 (4)	−0.06879 (2)	0.384221 (15)	0.02303 (8)	
Br2	0.13208 (4)	0.19690 (2)	0.442048 (15)	0.02540 (9)	

### Atomic displacement parameters (Å^2^)

**Table:**

	*U*^11^	*U*^22^	*U*^33^	*U*^12^	*U*^13^	*U*^23^
C1	0.0205 (15)	0.0199 (15)	0.0194 (17)	−0.0010 (12)	0.0011 (12)	−0.0021 (13)
C2	0.0196 (14)	0.0221 (16)	0.0182 (16)	−0.0003 (12)	0.0041 (12)	0.0006 (13)
C3	0.0170 (14)	0.0258 (17)	0.0271 (18)	−0.0032 (13)	−0.0012 (13)	0.0004 (14)
C4	0.0184 (14)	0.0291 (17)	0.0190 (17)	−0.0016 (13)	0.0000 (12)	−0.0022 (13)
C5	0.0221 (15)	0.0270 (18)	0.0201 (17)	−0.0048 (13)	0.0011 (12)	−0.0027 (14)
C6	0.0231 (15)	0.0201 (16)	0.0175 (17)	−0.0008 (12)	0.0038 (13)	−0.0014 (13)
C7	0.0297 (17)	0.0248 (17)	0.0212 (17)	−0.0031 (14)	0.0064 (13)	−0.0039 (14)
C8	0.0315 (18)	0.0267 (18)	0.0244 (19)	−0.0003 (15)	0.0068 (15)	0.0035 (14)
C9	0.054 (2)	0.035 (2)	0.025 (2)	−0.0122 (18)	0.0112 (18)	−0.0017 (18)
C10	0.0352 (18)	0.0329 (19)	0.0177 (16)	0.0066 (16)	0.0071 (15)	0.0022 (14)
C11	0.048 (2)	0.0281 (19)	0.0158 (17)	0.0061 (16)	0.0029 (15)	−0.0047 (15)
C12	0.0373 (19)	0.0216 (17)	0.0203 (17)	0.0013 (14)	0.0008 (14)	−0.0007 (13)
N1	0.0179 (12)	0.0223 (13)	0.0162 (14)	−0.0015 (10)	0.0032 (10)	−0.0013 (11)
N2	0.0270 (15)	0.0254 (16)	0.044 (2)	−0.0011 (12)	−0.0020 (14)	−0.0030 (15)
N3	0.0224 (13)	0.0221 (14)	0.0165 (14)	0.0023 (11)	0.0015 (11)	−0.0030 (11)
N4	0.095 (3)	0.049 (2)	0.032 (2)	−0.031 (2)	0.017 (2)	−0.0050 (19)
Zn1	0.01815 (16)	0.02033 (17)	0.01577 (17)	−0.00049 (14)	0.00133 (14)	−0.00152 (15)
Br1	0.02130 (14)	0.02218 (16)	0.02562 (17)	−0.00458 (13)	0.00254 (13)	−0.00263 (13)
Br2	0.02787 (16)	0.02558 (16)	0.02274 (17)	0.00407 (13)	0.00031 (14)	−0.00736 (14)

### Geometric parameters (Å, °)

**Table:**

C1—N1	1.335 (4)	C7—H7	0.93
C1—C2	1.394 (4)	C8—C10	1.387 (5)
C1—H1	0.93	C8—C9	1.436 (5)
C2—C4	1.394 (5)	C9—N4	1.148 (5)
C2—C3	1.445 (5)	C10—C11	1.368 (5)
C3—N2	1.145 (5)	C10—H10	0.93
C4—C5	1.377 (5)	C11—C12	1.386 (5)
C4—H4	0.93	C11—H11	0.93
C5—C6	1.392 (4)	C12—N3	1.346 (4)
C5—H5	0.93	C12—H12	0.93
C6—N1	1.347 (4)	N1—Zn1	2.061 (3)
C6—H6	0.93	N3—Zn1	2.072 (3)
C7—N3	1.336 (4)	Zn1—Br2	2.3369 (5)
C7—C8	1.392 (5)	Zn1—Br1	2.3471 (5)
			
N1—C1—C2	121.9 (3)	N4—C9—C8	178.4 (5)
N1—C1—H1	119.1	C11—C10—C8	118.4 (3)
C2—C1—H1	119.1	C11—C10—H10	120.8
C4—C2—C1	119.3 (3)	C8—C10—H10	120.8
C4—C2—C3	122.2 (3)	C10—C11—C12	119.3 (3)
C1—C2—C3	118.5 (3)	C10—C11—H11	120.3
N2—C3—C2	177.1 (4)	C12—C11—H11	120.3
C5—C4—C2	118.5 (3)	N3—C12—C11	122.4 (3)
C5—C4—H4	120.8	N3—C12—H12	118.8
C2—C4—H4	120.8	C11—C12—H12	118.8
C4—C5—C6	119.3 (3)	C1—N1—C6	118.9 (3)
C4—C5—H5	120.4	C1—N1—Zn1	118.4 (2)
C6—C5—H5	120.4	C6—N1—Zn1	122.6 (2)
N1—C6—C5	122.2 (3)	C7—N3—C12	118.6 (3)
N1—C6—H6	118.9	C7—N3—Zn1	122.0 (2)
C5—C6—H6	118.9	C12—N3—Zn1	119.1 (2)
N3—C7—C8	121.4 (3)	N1—Zn1—N3	100.85 (11)
N3—C7—H7	119.3	N1—Zn1—Br2	111.60 (8)
C8—C7—H7	119.3	N3—Zn1—Br2	108.98 (8)
C10—C8—C7	119.9 (3)	N1—Zn1—Br1	105.62 (8)
C10—C8—C9	120.4 (3)	N3—Zn1—Br1	105.82 (8)
C7—C8—C9	119.7 (3)	Br2—Zn1—Br1	121.841 (19)
			
N1—C1—C2—C4	1.2 (5)	C8—C7—N3—C12	−0.2 (5)
N1—C1—C2—C3	−177.2 (3)	C8—C7—N3—Zn1	174.7 (3)
C1—C2—C4—C5	−1.1 (5)	C11—C12—N3—C7	1.2 (5)
C3—C2—C4—C5	177.2 (3)	C11—C12—N3—Zn1	−173.9 (3)
C2—C4—C5—C6	0.1 (5)	C1—N1—Zn1—N3	−82.3 (3)
C4—C5—C6—N1	1.0 (5)	C6—N1—Zn1—N3	92.9 (3)
N3—C7—C8—C10	−1.1 (5)	C1—N1—Zn1—Br2	162.1 (2)
N3—C7—C8—C9	177.6 (3)	C6—N1—Zn1—Br2	−22.7 (3)
C7—C8—C10—C11	1.3 (5)	C1—N1—Zn1—Br1	27.7 (3)
C9—C8—C10—C11	−177.3 (4)	C6—N1—Zn1—Br1	−157.1 (2)
C8—C10—C11—C12	−0.3 (6)	C7—N3—Zn1—N1	−77.5 (3)
C10—C11—C12—N3	−0.9 (6)	C12—N3—Zn1—N1	97.4 (3)
C2—C1—N1—C6	−0.1 (5)	C7—N3—Zn1—Br2	40.1 (3)
C2—C1—N1—Zn1	175.3 (2)	C12—N3—Zn1—Br2	−145.1 (2)
C5—C6—N1—C1	−1.0 (5)	C7—N3—Zn1—Br1	172.7 (2)
C5—C6—N1—Zn1	−176.2 (2)	C12—N3—Zn1—Br1	−12.4 (3)

## References

[bb1] Bruker (2001). *SMART*, *SAINT* and *SADABS* Bruker AXS Inc., Madison, Wisconsin, USA.

[bb2] Farrugia, L. J. (1997). *J. Appl. Cryst.* **30**, 565.

[bb3] Farrugia, L. J. (1999). *J. Appl. Cryst.* **32**, 837–838.

[bb4] Li, X.-H., Wu, H.-Y. & Hu, J.-G. (2004). *Acta Cryst.* E**60**, m1533–m1535.

[bb5] Sheldrick, G. M. (2008). *Acta Cryst.* A**64**, 112–122. 10.1107/S0108767307043930 18156677

[bb6] Steffen, W. L. & Palenik, G. J. (1976). *Acta Cryst.* B**32**, 298–300.

[bb7] Steffen, W. L. & Palenik, G. J. (1977). *Inorg. Chem.* **16**, 1119–1127.

